# Surface Modified Multifunctional and Stimuli Responsive Nanoparticles for Drug Targeting: Current Status and Uses

**DOI:** 10.3390/ijms17091440

**Published:** 2016-08-31

**Authors:** Panoraia I. Siafaka, Neslihan Üstündağ Okur, Evangelos Karavas, Dimitrios N. Bikiaris

**Affiliations:** 1 Laboratory of Polymer Chemistry and Technology, Department of Chemistry, Aristotle University of Thessaloniki, Thessaloniki 54124, Macedonia, Greece; siafpan@gmail.com; 2 Department of Pharmaceutical Technology, School of Pharmacy, Istanbul Medipol University, Beykoz 34810, Istanbul, Turkey; nustundag@medipol.edu.tr; 3 Pharmathen Company, Dervenakion Str. 6, Attiki 15351, Greece; ekaravas@pharmathen.gr

**Keywords:** multifunctional nanocarriers, surface modification, drug delivery, cell targeting, stimuli responsive, toxicity

## Abstract

Nanocarriers, due to their unique features, are of increased interest among researchers working with pharmaceutical formulations. Polymeric nanoparticles and nanocapsules, involving non-toxic biodegradable polymers, liposomes, solid lipid nanoparticles, and inorganic–organic nanomaterials, are among the most used carriers for drugs for a broad spectrum of targeted diseases. In fact, oral, injectable, transdermal-dermal and ocular formulations mainly consist of the aforementioned nanomaterials demonstrating promising characteristics such as long circulation, specific targeting, high drug loading capacity, enhanced intracellular penetration, and so on. Over the last decade, huge advances in the development of novel, safer and less toxic nanocarriers with amended properties have been made. In addition, multifunctional nanocarriers combining chemical substances, vitamins and peptides via coupling chemistry, inorganic particles coated by biocompatible materials seem to play a key role considering that functionalization can enhance characteristics such as biocompatibility, targetability, environmental friendliness, and intracellular penetration while also have limited side effects. This review aims to summarize the “state of the art” of drug delivery carriers in nanosize, paying attention to their surface functionalization with ligands and other small or polymeric compounds so as to upgrade active and passive targeting, different release patterns as well as cell targeting and stimuli responsibility. Lastly, future aspects and potential uses of nanoparticulated drug systems are outlined.

## 1. Introduction

Nanocarriers (NCs) involve different types of nanomaterials (NMs) being used in pharmaceutical technology in order to allow drugs to be delivered throughout the body tissue. Nonetheless, sizes ranging from 1 to 1000 nm have been approved for NCs whereas nanoparticles (NPs) range between 1 and 100 nm in size [1]. NMs have emerged as promising carriers in the pharmaceutical and medical field in the role of imaging agents or drug carriers in view of their numerous beneficial properties, having a large surface volume ratio, biological mobility, enhanced tissue penetration, drug protection against degradation, and control of the drug release rate allowing the reduction of drug administration doses [2].

The first attempt of using targeted NCs in medicine was proposed in the 19th century by the scientist Paul Elrich which describes active targeting of nanosized drug delivery systems. In the 1960s, Professor Peter Paul Speiser focused on developing nanoparticulate systems for vaccination processes [3]. Since then, marketed products based on nanotechnology are available while others are under clinical trial. 

Recent progress in nanotechnology has had a major impact on cancer diagnosis and therapy [4,5] given that cancer is the main cause of death in current generations; only in 2016, 1,685,210 new cancer cases and 595,690 cancer deaths are projected to occur in the United States [6]. Chemotherapy using anticancer drugs is in most cases the main treatment along with surgeries, but often toxic side effects are responsible for halting the treatment since these effects cause severe damage to the immunological system. Along with neoplastic diseases, developing novel therapies for heart diseases, strokes and other less risky disorders is now more significant than ever. Actually, promising efforts are being directed towards producing drug delivery formulations for the administration of therapeutic agents with minimal side effects and selectively target the specific-tissue.

**Figure 1:**
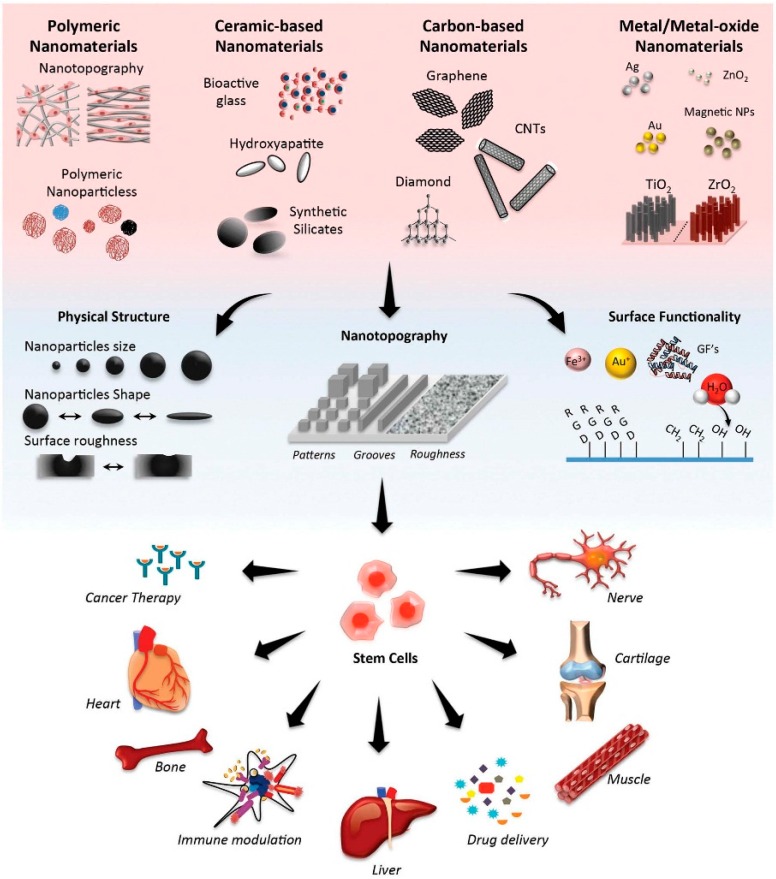
Representative nanoparticles that are used as appropriate drug delivery vehicles and their interactions with stem cells [13].

Over time, several versatile and intelligent nanocarriers have been prepared as advanced drug delivery carriers, including inorganic nanoparticles, such as gold, silver and silica, carbon based materials like nanotubes and graphenes, lipid-based vehicles like liposomes and lipid particles, polymeric based systems like nanoparticles, micelles, dendrimers, virus-like materials, hydrogels and emulsions (Figure 1) [7,8,9,10,11,12,13,14].

In terms of drug delivery, the nanoparticles’ characteristics are of particular importance. Nanoparticles with an external diameter ranging between 50 and 400 nm can be applied for anticancer drug targeted delivery while similar sizes are assumed as critical for ocular formulations. As follows, the circulation of nanocarriers in the human body and their uptake by different tissues depends on the targeted tissue. Nanoparticles smaller than 1 nm are effective to pass the blood–brain barrier while continuous capillaries like those found in muscles, lungs and skin are permeable by nanoparticles with sizes of about 6 nm. Bigger nanoparticles in the range of 40–60 nm are appropriate to exit the fenestrated capillaries of several organs like kidney, intestine and in some endocrine/exocrine glands. Even more, the largest nanoparticles (>600 nm) can agglomerate in the liver and spleen as well as in bone marrow [15]. Furthermore, nanoparticles with a positive surface charge are capable of being attached more rapidly to the negatively charged surface of cells and thus can be endocytosed easily from them.

Nowadays, researchers focus on NCs with a view to maintain chemical reactivity and targeting. In the design of smart NCs, several requirements exist: first, the targeted delivery of an active compound with penetration through membranes and endocytosis to reach the target site; second, the ability of the carrier to escape from biological protective mechanisms such as opsonization and RES clearance; and finally to communicate and recognize the environmental changes. Therefore, for smart NCs, modification and functionalization of their surface with moieties which are responsive to a range of different stimuli is beneficial. These stimuli can be endogenous factors such as redox, enzyme, and pH, or exogenous factors such as light, ultra sound, and magnetic fields, or to temperature that can either be endogenous or exogenous [16]. Surface functionalization of NCs such as polymeric nanocapsules and nanoparticles, lipid nanoparticles and inorganic materials can determine the half-life time, biocompatibility, stimuli-responsiveness and therapeutic scope [17]. Moreover, coating using both natural and synthetic polymers also demonstrated intriguing properties [18].

This review focuses on the application of different nanoparticles as drug carriers for cell and tissue targeting. Emphasis was given to various surface functionalizing agents of these nanoparticles, which is necessary in order to achieve long blood circulation, communicate with the human body recognizing any environmental changes and responding to these changes, and also delivering the drug to recognized cell targets.

## 2. Types of Nanocarriers

The classes of nanocarriers listed herein are the most intriguing and frequently used carriers for pharmaceutical applications. The nanocarriers included in this review fall into four main categories, which are inorganic and organic NPs, lipid based NCs and polymeric NPs, which all of them currently poised to have an impact as part of the ongoing research on smart treatments.

### 2.1. Carbon-Based Nanoparticles

Carbon-based nanoparticles such as carbon nanotubes, fullerenes, grapheme, carbon dots and nanodiamonds represent a prominent area of nanoparticle research and application as drug delivery materials [19]. Carbon nanotubes and graphene are both low-dimensional sp^2^ carbon nanoparticles exhibiting many unique physical and chemical properties and have been extensively explored as potential drug delivery carriers in recent years.

#### 2.1.1. Carbon Nanotubes (CNTs)

Since the discovery of CNTs in 1991 by Sumio lijima, the revolutionary development of these nanotubes for the application of nanomedicine has emerged as one of the most interesting fields, which has increased exponentially in size in recent years. These nanotubes can be either single-walled (SWCNTs) or multi-walled (MWCNTs), and they are highly ordered, pseudo one-dimensional carbon allotropes. SWCNTs comprise of a rolled-up single layer of graphite cylinder with a tube diameter of 0.3–2 nm, whereas MWCNTs are multiple concentric cylindrical shells of graphite sheets with an interlayer distance of approximately 0.36 nm with diameters of 2–100 nm. Because they are hollow and with dimensions smaller than the blood cells, they are ideal drug candidates. Furthermore, CNTs are capable of penetrating into cells and delivering the therapeutic molecules at the targeted site [20]. Since most of drugs have aromatic groups, these can be absorbed inside or outside of CNTs through π−π noncovalent stacking.

For a drug delivery system to be investigated and approved, it should be first studied how the used vehicles interact with body cells and accumulate in different body organs after its systemic administration should be first studied. The toxicity of carbon nanotubes is still a discussed issue and it is believed that pristine CNTs have some cytotoxicity and cause inflammation to human organs [21]. In a recent review, the role of CNT characteristics like their dimensions and surface properties in their toxicity was highlighted [22]. However, the scientific community does not have clear and strong evidence of whether carbon nanomaterials are toxic or not, and for this reason further research is needed in the near future before their safe application in a clinical setting can be ensured [23,24]. Thus, the safe use of CNTs in biological applications remains an open question. On the contrary, functionalized CNTs (f-CNT) exhibit low accumulation and can be excreted from the human body reducing their toxicity [25,26]. Up until now, much research has been done in order to study the biological behaviour of f-CNT in vitro and in vivo. The current results indicate that f-CNTs may have some biocompatibility as well as lower cytotoxicity [27,28,29,30,31]. The observed cytotoxicity was correlated with the functionalization extent of f-CNTs and the chemical structure of used groups that revealed a concentration dependent on cytotoxicity profile [32].

The functionalization of the CNTs surface walls can alter their physiological and biological properties while improving the solubility of anticancer molecules drugs in order to efficiently target tumour [33]. The current aim for CNTs’ functionalization is to prepare water-soluble materials with high biocompatibility for high treatment efficacy and minimum side effects [34,35,36,37]. CNTs have been functionalized with β-cyclodextrin [38], betunilic acid [39], glucosamine [40], aptamer and piperazine-polyethylenimine derivative [41], as well as with many other organic materials in order to be used as appropriate nanoplatforms for the entrapment and delivery of several drugs. Cationically functionalized CNTs with polyethyleneimine (PEI) and with pyridinium were also investigated for delivery of siRNA [42] and for melanoma treatment [43]. In a similar approach, MWCNTs have been modified with PEI and also covalently conjugated with fluorescein isothiocyanate (FI) and hyaluronic acid (HA) [44]. These functionalized MWCNTs were used as nanocarriers of doxorubicin (DOX) appropriate for targeted cancer cells overexpressing CD44 receptors. It was found that these MWCNT/PEI-FI-HA/DOX formulations have high drug loading (72%) and are water soluble. In vitro release studies showed that the drug release at pH 5.8 is higher than at pH 7.4. Furthermore, it was reported that the prepared material has good biocompatibility and can inhibit cancer cells’ growth. PEI was also covalently grafted with MWCNT to prepare a multifunctional platform with simultaneous conjugation of fluorescein isothiocyanate (FITC) and prostate stem cell antigen (PSCA) monoclonal antibody (mAb) [45]. The prepared functionalised nanocarrier showed high biocompatibility, and it was proved that the antibody conjugation improves the cellular uptake ability of the complex by PSCA-overexpressed tumour cells. The results were also interesting after in vivo anti-cancer tests using PC-3 tumour-bearing mice. It was found that the prepared CNT-PEI(FITC)-mAb complexes can act as drug targeting delivery systems inhibiting tumour growth.

**Figure 2:**
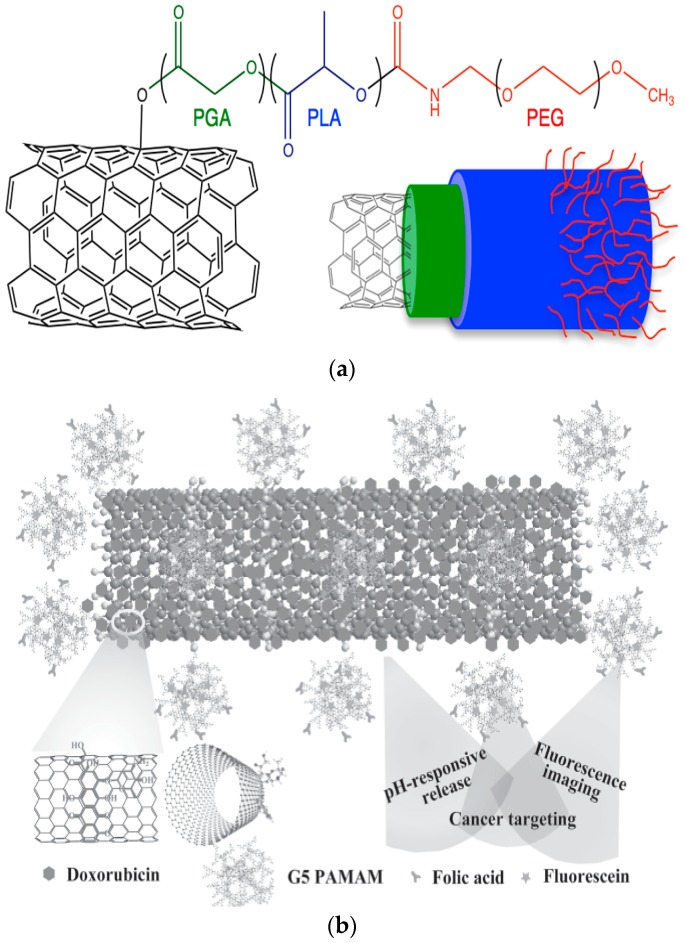
(**a**) Surface modification of carbon nanotubes (CNTs) with biocompatible PEGylated polymers [46]; (**b**) Schematic illustration of folate-targeted and fluorescently labeled multifunctional multiwalled carbon nanotubes (MWCNTs) for targeted and pH-responsive delivery of doxorubicin (DOX) to cancer cells [48].

PEGylation is also a used procedure for surface modification of CNTs, using PEG alone or in conjugation with other biocompatible polymers (Figure 2a) [46]. Such novel PEG surface modified SWCNTs with nucleic acids have been recently prepared [47]. 5′-Pyrene was also used to immobilize oligonucleotides on the SWCNT surface. Based on MTT assays, it was found that both f-SWCNTs and their hybrids with oligonucleotides appeared to have low cytotoxicity against different cells like HeLa, KB-3-1 and KB-8-5. A similar drug-targeted delivery system of DOX consisting of PEG functionalized and folic acid conjugated SWCNTs was also prepared, for targeted killing of breast cancer cells [48]. From DOX-release studies, it was found that DOX can be released only at pH 4.0, which is close to the tumour environment pH, while at pH 7.4 it remained entrapped in the conjugated system. Furthermore, DOX can be released sustainably from this system for a prolonged time of 3 days. In another work, PEGylated-folate-CNTs was prepared as an appropriate nanocarrier for DOX drug delivery [29]. From in vitro and in vivo studies, it was found that this novel PEGylated-folate-CNTs system could be an effective drug (DOX) delivery system improving simultaneously the biodistribution and pharmacokinetic properties of anticancer drug.

Folic acid (FA) is found to be the most used compound for drug targeting applications conjugated with anticancer drugs or nanocarriers. In a recent study, poly(amidoamine) dendrimers (PAMAM), which were amine-terminated and also functionalised with FA and a fluorescein agent like isothiocyanate, were covalently bonded into acid-treated MWCNTs (Figure 2b) [49]. This multifunctional system was proposed to act simultaneously as a drug targeted and pH-responsive system, delivering DOX anticancer drug into cancer cells. The prepared complexes displayed effective therapeutic efficacy and it was proved that they have high affinity to FA receptors and thus were able to target cancer cells inhibiting their growth. Except dendrimers, natural polymers were also used for functionalization of CNTs. In such study, a pH and simultaneous thermo sensitive nanogel was synthesized by encapsulating SWCNTs with chitosan and PNIPAAm (CS/PNIPAAm/CNT) in order to prepare stimuli-responsive drug delivery systems with enhanced tumour-targeting drug transportation [50]. The prepared nanoparticles contain 43% DOX anticancer drug, while the drug can be released faster at 40 °C than at 25 °C as well as faster at pH 5.0 than at pH 7.4.

Another extensively functionalizing agent of CNTs is hyaluronic acid. Multifunctional SWCNTs have been prepared with distearoylphosphatidylethanolamine-hyaluronic acid (DSPE-HA) in order to reduce cytotoxicity of SWCNTs and also to act as targeting vehicles to CD44-overexpressing MDR cancer cells [51]. This strategy upgraded the intracellular epirubicin (EPI) drug delivery and overcame the multidrug resistance (MDR) of cancers cells. The results showed that the synthesized EPI/SWCNTs/DSPE/HA multifunctional system had insignificant toxicity and increased intracellular delivery and retention of epirubicin indicating that the prepared system could be a promising nanocarrier for anticancer drug delivery. In a recent study, SWCNTs were conjugated with HA while gadolinium (Gd) and DOX drug were also added in order to develop a redox-sensitive system [52]. From in vitro drug release studies, it was found that DOX was released much faster under reducing conditions. Furthermore, confocal microscopy images showed that the prepared system was capable of simultaneously delivering SWCNTs and thus DOX into Michigan Cancer Foundation-7 cells via HA receptor-mediated endocytosis.

#### 2.1.2. Graphene (GF) and Graphene Oxide (GO)

Graphene (GF) and graphene oxide (GO) are nanosheets consisting of two-dimensional single monoatomic layers of sp^2^ hybridized carbon atoms. They can be easily synthesized and GF is a hydrophobic nanomaterial while GO is an oxidized form of GF. GO due to its chemical treatment contains carboxyl, hydroxyl and epoxy groups and, thus, its surface can be easily functionalized with several small molecules or with polymers. Both have high specific surface area, which is ideal for high drug loading via π–π stacking and hydrophobic or electrostatic interactions [53]. However, GF has the same problem as pristine CNTs and, due to its hydrophobicity, is very difficult to clear from the human body. In a current review, all studies about GF toxicity were summarized and it was concluded that their toxicity directly stems from the graphene surface and the kind of functionalization or used coatings, the administration doses and routes as well as from the exposure time on different cells [54]. The toxicity mechanism of GF is based to the formation of reactive oxygen species in target cells. Furthermore, it was found that the hydrophilic form of GF like GO can form a stable colloid dispersion and thus avoids aggregation, which is the key to easy removal from the application site. However, even though GO are more hydrophilic, some early studies showed that these nanosheets exhibited time and dose-dependent cytotoxicity, and managed to enter into the cytoplasm and nucleus, decreasing cell adhesion, and inducing cell floating and apoptosis [54,55,56,57,58]. Thus, surface functionalization can play a major role in providing GO appropriate vehicles for several therapeutics like drug molecules, and especially anticancer drugs, DNA, genes, antibodies, antibacterial, proteins, etc. [59,60,61,62,63,64,65].

Pegylated GO nanoparticles treated with polyethylenimine and other polymers like chitosan, poly(*N*-isopropylacrylamide), polyacrylates, poly-l-lysine, poly(sodium 4-styrenesulfonate), dextran, pluronic, proteins, with small molecules like folic acid and cyclodextrins have been broadly applied as drug carriers [66,67,68,69,70,71,72,73,74,75]. In such work, a multifunctional system consisting of PEI/poly(sodium-4-styrenesulfonates) (PSS)/GO was prepared in order to evaluate its use as a delivery agent of adriamycin (ADR) along with miR-21 targeted siRNA in cancer drug resistance [76]. Based on the contacted cell studied, it was proved that this multifunctional system significantly enhanced the accumulation of ADR in MCF-7/ADR cells and simultaneously had higher cytotoxicity than free ADR. Similarly, multifunctional nanosheets consisted from HA/GO/pluronic with targeted chemo-photo thermal properties of mitoxantrone (MIT) have been prepared in order to overcome multidrug resistance with in vitro release studies showing that these nanosheets were internalized into MCF-7/ADR cells via receptor-mediated endocytosis. (MDR) [77]. Furthermore, in vivo studies in mice confirmed that these systems were the most effective among all MIT formulations. Recently, a different multifunctional GO system with PEI, isothiocyanate (FITC) as fluorescein agent and PEG-bonded lactobionic acid (LA) was mentioned so as to target the hepatocarcinoma cells [67]. Finally, PEI was acetylated and DOX was nanoencapsulated establishing that these nanocarriers have good cell viability and can specifically target cancer cells overexpressing asialoglycoprotein (ASGPR) receptors and exert a growth inhibition effect on the cancer cells.

**Figure 3:**
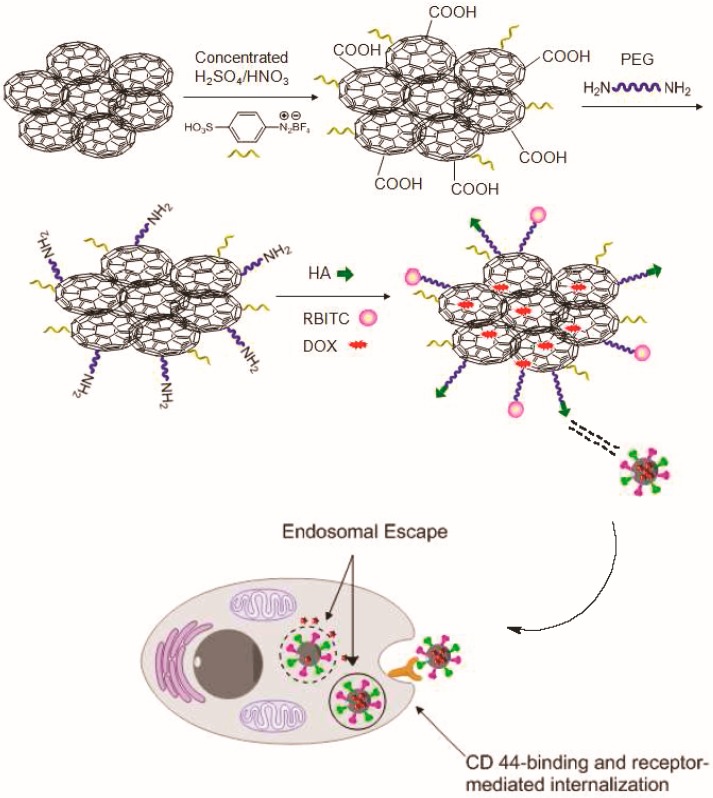
Synthetic path of DOX loaded HA-Q-G-RBITC multifunctional nanosheets and HA-Mediated Endocytosis [78].

Hyaluronic and folic acid were also used for graphene functionalization. In such a system, HA andrhodamine B isothiocyanate were introduced into Q-Graphene preparing a dual function nanocarrier, which was appropriate for targeted drug delivery and fluorescence imaging (Figure 3) [78]. DOX was loaded onto the Q-GF surface via π−π stacking. It was found that these nanoparticles were effective nanoplatforms for targeting tumour cells since were effectively internalized by HA-positive A549 cells through HA-mediated cellular endocytosis.

In another study, GO were functionalized with HA (HA-GO) and loaded with 45 wt% mitoxantrone (MIT) [77]. The results revealed that these nanosheets exhibited pH-sensitive responses to the tumour environment and enhanced internalization. Graphene quantum dots (GQDs) conjugated with FA ligand have been also prepared and loaded with DOX [79] reporting that the prepared nanosheets can effectively discriminate cancer cells from normal cells and efficiently deliver the drug to targeted cells. Moreover, it was observed that these nanosheets are rapidly and specifically internalized by HeLa cells via receptor-mediated endocytosis.

GF or GO can be used either alone or in hybrids or mixtures with other nanoparticles. In a recent work, polyglycerol-g-polycaprolactone copolymers were grafted on mesoporous silica-coated magnetic graphene oxide complex [80]. DOX was loaded on this carrier as an anticancer drug and from in vitro DOX release studies demonstrated a controlled pH responsive behaviour. Likewise, nanoparticles functionalized with chitosan magnetic graphene (CMG) have been prepared by Wang et al. [81] and loaded also with DOX. It was came out that DOX released faster at pH 5.1 compared to normal pH 7.4, and was more effective (IC_50_ = 2 mM) in killing A549 lung cancer cells than free DOX (IC_50_ = 4 mM). Besides, functionalized GO–gold nanoparticle nanocomposites for DNA aptamer (Apt) targeting were also recently formulated encapsulating DOX [82] showing heat-stimulated and sustained release characteristics. Further, in vitro cell cytotoxicity experiments showed that such a combined therapy had the highest rate of tumour cell death compared to single chemotherapy. Another interesting finding was that modification with aptamers can significantly enhance the accumulation of these composites within cancer cells.

#### 2.1.3. Nanodiamonds (NDs)

Nanodiamonds are 2–10 nm diameter carbon nanoparticles of a truncated octahedral structure. Due to their cheap and large-scale synthesis, unique characteristics and optical advantages, NDs have been studied as a promising, alternative material for biological and medical applications. Additional advantages of NDs include large surface area, enhanced biocompatibility, good mechanical strength, high surface functionality and colloidal stability. However, for most biomedical applications including drug delivery, the precise control of their surfaces via functionalization is necessary [83,84,85,86,87]. It has been pointed out that NDs are not used neat but are conjugated with biocompatible polymers and drugs, similarly to previously reported cases with CNTs and GOs focusing on cancer treatment [84,88,89,90,91,92].

### 2.2. Inorganic Nanoparticles

Lately, metal nanoparticles have been greatly examined as appropriate carriers for biomedical applications. The fact that these nanoparticles can be used in diagnosis and drug delivery due to their unique properties such as small size, extremely high surface area, high reactivity to the living cells, ability for functionalization, etc., classify them as an important category for study. For biomedical applications, the widely used metal nanoparticles are gold, silicon oxide, silver, titanium oxide and iron oxide [93].

#### 2.2.1. Gold Nanoparticles (GNPs, AuNPs)

Gold nanoparticles (AuNPs, GNPs) in sizes of 1–100 nm are extensively used for drug and gene delivery since they are inert and have lower toxicity than other metal nanoparticles [94,95,96,97]. Despite these features, GNPs’ surface functionalization is mandatory in order to apply them to specific disease areas and allow them to selectively interact with cells [98,99,100].

**Figure 4:**
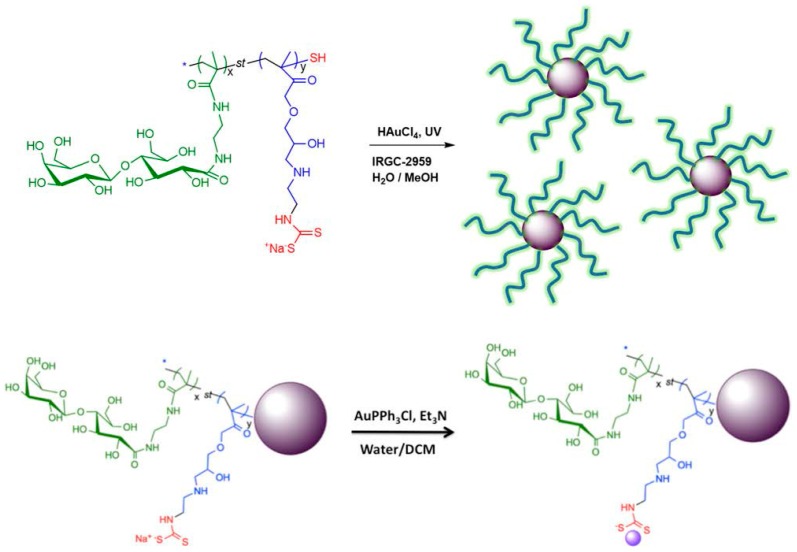
Synthesis of polymer functionalized gold nanoparticles and their conjugation to the anticancer drug Au(1)PPh3 [104].

Several methods are concerned with the functionalization of GNPs with different compounds like PEG, DNA and RNA, peptides, antibodies, lipids, and also small drug molecules to increase their adhesion and interactions with biological molecules and cells acting as targeted drug-carriers [99,101,102,103,104,105,106]. In such study, glyco-dithiocarbamate (DTC) copolymers were synthesized by reversible addition-fragmentation chain transfer polymerization (RAFT) and subsequently used to prepare glyconanoparticles and conjugated glyconanoparticles with the anticancer drug gold(I) triphenylphosphine (Figure 4) [104]. From cytotoxic studies, it was evidenced that these glycopolymer-drug conjugated AuNPs had approximately four times higher cytotoxicity toward HepG2 cells overexpressing asialoglycoprotein (ASGPR) as compared to ASGPR-deficient Hela cells.

In a recent study, it was pointed out that the most critical step in the penetration process is potentially the fusion of GNPs with lipid bilayers and this is possible only when GNPs have a core diameter below a critical size [107]. Another research used functionalized An-PEG-DOX-AuNPs loaded with DOX to target glioma cells [108]. The advantage of this nanocarrier was that by functionalizing with angiopep-2, a specific ligand of low density lipoprotein receptor-related protein-1, which could mediate the system to penetrate the blood–brain barrier and target glioma cells, DOX was specifically delivered to glioma cells and expanded the median survival time of glioma-bearing mice. In a similar manner, thrombin-binding aptamer-conjugated gold nanoparticles (TBA-AuNPs) have been synthesized and it was reported that they can effectively inhibit thrombin activity toward fibrinogen [109]. AuNPs can also form hybrid nanocomposites with other nanoparticles like fullerene. Such C60@Au nanocomposites were prepared and functionalised with PEG5000 using also a pH cleavable hydrazone bond [110]. This system was loaded with DOX and can be used for targeting tumour tissues.

Even though AuNPs have been applied as potential drug delivery nanocarriers, their safe use and cytotoxicity should be investigated. However, many of mentioned studies in literature, based on a large variety of experimental conditions and different protocols, have led to completely different results concerning the safe use of GNPs in human applications [111]. The potential toxic impact of AuNPs may be multifaceted and hard to predict [112]. It was mentioned that particles size and the surface functionality of GNPs play a crucial role in determining genotoxic-, mutagenic- or cell toxic effects [113]. Data obtained from a host of methods including CCK-8, MTT assay and other analytical techniques showed no apparent cytotoxicity of GNPs in cancer or healthy cells [114]. In a recent work, the effects of GNPs were investigated in vitro on Balb/3T3 mouse fibroblasts [115] showing that after cell explosion for 72 h of GNPs with 5 and 15 nm particle size diameters, only those with 5 nm at concentration ≥50 μM showed some cytotoxic effects. After 24 h of exposure time, multivesicular double membrane bodies loaded with GNPs were observed indicating autophagosomes’ formation. Furthermore, GNPs were shown to damage the cytoskeleton organization. In another study, neat GNPs with diameters of 3–100 nm were injected intraperitoneally into BALB/C mice at a dose of 8 mg/kg/week [116] revealing that GNPs with diameters ranging from 8 to 37 nm induced severe sickness in mice. From the above, it seems that GNPS adsorption, metabolism, and mainly their excretion is rather unclear.

#### 2.2.2. Silver Nanoparticles (AgNPs)

Silver nanoparticles show significant antimicrobial properties and for this reason are applied in wound dressings, dental hygiene, and treatment of eyes [117,118]. Evidently, silver is well known to exhibit strong toxicity in a wide range of micro-organisms, and, thus, silver-based formulations have been extensively used in antibacterial applications [119]. Except for the aforementioned application, the effectiveness of surface-functionalization in specific targeting of either tumour cells or immune cells in vivo was found to be critical for drug delivery systems [120]. Functionalized AgNPs coated with maleimide have been prepared as cross-linkers for the preparation of gelatin based hydrogels [121]. The low cytotoxicity of the hydrogels showed their possible utility as potential drug delivery systems or in tissue engineering. AgNPs coated with galactose and mannose agents with neuronal-like cells and hepatocytes also displayed low toxicity compared to AgNPs coated by glucose, ethylene glycol or citrate [122]. In a recent research a drug-delivery system based on AgNP achieved the simultaneous intracellular delivery of both DOX and alendronate (Ald) anticancer drugs in order to improve the anticancer therapeutic of the formulation [123]. It was revealed that the above AgNPs’ nanoparticles loaded with both drugs had significantly higher anti-cancer activity in vitro than either Ald or Dox alone.

#### 2.2.3. Iron Oxide Nanoparticles (IONPs)

Iron oxides were mainly used as magnetic nanoparticles for many biological applications. On the contrary, for specific uses and drug targeting, they need to fulfil special criteria with respect to particle size and their distribution, surface functionalization with appropriate ligands, biocompatibility, etc. [124]. A current review extensively discussed the applications of IONPs for cancer therapy and diagnosis and, especially in MRI, drug delivery, hyperthermia and other interesting applications [124]. It is clear that previously various covalent and non-covalent ligands have been applied for the functionalization of IONPs and these functionalised nanoparticles have been studied as appropriate drug nanocarriers [125,126,127,128,129]. According to these studies, three main coupling strategies for functionalization and linking IONPs with active agents were proposed: (i) surface modification of IONPs with reactive amine groups; (ii) chemical modification with fluorescent additives; and (iii) functionalization of carboxyl groups for enzyme immobilization. The combination of drug target delivery, controlled drug release and simultaneous MRI observation makes IONPs an excellent drug nanocarrier.

In an up to date study, it was reported that the therapeutic effects of breast cancer magnetic hyperthermia could be strongly enhanced by the combination of functionalized MF66 oxide nanoparticles with Nucant multivalent pseudopeptide (N6L), DOX or both (MF66-N6LDOX) and magnetic hyperthermia [130]. Stable aqueous suspension of IONPs with a size of 20 nm that linked with cinnamaldehyde, glycine and pluronic have been also examined for potential application in drug delivery and hyperthermia in breast cancer [131]. It was indicated that these nanoparticles decreased the breast cancer cells’ growth and this effect was directly dependent on the used dose.

### 2.3. Mesoporous Nanoparticles (MSN or MSNPs)

In contrast to nonporous nanoparticles, mesoporous nanoparticles (MSN or MSNPs) have a solid framework with particle size diameters of 50–300 nm, an interior porous structure and a narrow pore size of 2–6 nm, large pore volume (0.5–2.0 cm^3^/g), high surface area (500–3000 cm^2^/g) and ordered pore networks. They are nontoxic in nature, can be easily functionalized, have large loading capacity and good biocompatibility. Furthermore, mesoporous silica materials have also displayed structural stability in storage [14]. IUPAC has classified MSNPs as porous solid materials according to their pore diameter; if the diameter of pores is <2 nm, they are called “microporous” in contrast, if the diameter of pores is >50 nm, they are called “macroporous” and materials with a pore diameter between 2 and 50 nm are called “mesoporous” [132]. All the above characteristics of MSNs make them ideal nanocarriers for drug delivery applications especially for anticancer agents which have poor water solubility [133]. MSN have also been investigated as gene delivery carriers, proteins, enzymes, etc. [132]. Studies have shown that MSN with diameters below 300 nm are favourable for delivery of therapeutic compounds through endocytosis, but in the case of larger particles, phagocytosis is the predominant mechanism of cell uptake.

The main drawback of MSNPs is the resulted haemolysis, which is attributed to the surface silanol groups which can interact with the surface of the phospholipids of the red blood cell membranes as well as the metabolic changes induced by MSNPs leading to melanoma promotion. Nonetheless, these problems can be overcome since MSNPs involve the modification of silanol groups with a wide range of organic functional groups, minimizing opsonisation and thus leading to rapid clearance of MSNPs [134]. Among other benefits, functionalization can produce nanocarriers with stimuli-responsive drug release capability enhancing the efficiency and minimising the side effects of anti-tumour drugs for cancer therapy [135].

Two different approaches have been considered concerning preparation of MSN with controlled and drug targeting properties. The first approach involves the use of ligands attached to the surface of MSNPs for specific retention and uptake by the targeted disease cells. To this aim, several compounds were chosen to bind surface molecules or receptors overexpressed in diseased organs, tissues, cells or organelles. Typical ligands include peptides, antibodies, biocompatible polymers [136] such as aliphatic polyesters and PEG, proteins, aptamers, saccharides and small molecules such as vitamins or folic and hyaluronic acids. Another approach, and maybe the most important, is the so-called “capping” or “gating” approach. According to this, small molecules can be attached at the pore opening, closing the pore gates and thus preventing release of the drug stored inside the pores. Such MSNs are able to respond to internal and external stimuli carrying out controlled release of anticancer drugs and have been developed as very effective nanocarriers. This stimuli can be endogenous factors such as redox, enzyme, and pH, or exogenous factors such as light, ultra sound, and magnetic fields, or temperature that can either be endogenous or exogenous [14,16]. For this reason, appropriate nanovalves can be attached to the pore gates in order to control when and how these pores will be opened and closed inducing the stimuli response procedure. Cargo molecules and mainly anticancer drugs can be loaded in these pores.

**Figure 5:**
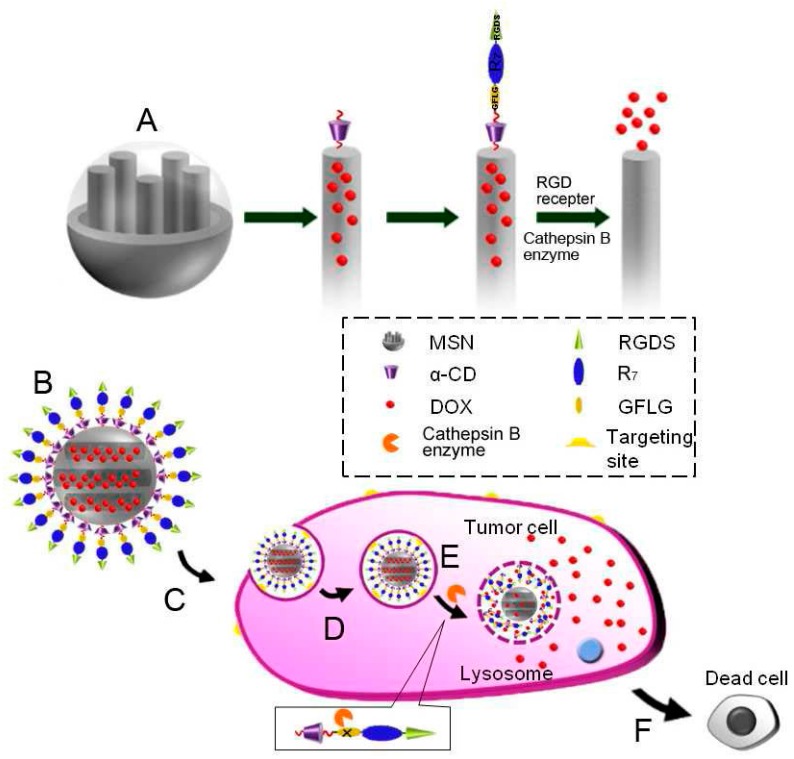
(**A**) Functionalization procedure of MSNs; (**B**) Drug-loaded MSNs under physiological condition; (**C**) RGDS-targeted to the tumour cell; (**D**) Endocytosis into specific tumour cell; (**E**) CathepsinB enzyme-triggered drug release in cytoplasm; (**F**) Apoptosis of the tumour cell [146].

PH-sensitive activation of MSNPs is of particular interest to prepare appropriate carriers that can be autonomously activated in vitro and in vivo since tumour cells have a lower pH environment due to hypoxic conditions and thus an anticancer loaded drug can be released into cancer cells according to this procedure. With the passage of years, several strategies for a controlled release mechanism that responds to low pH environments were investigated. In such an example, DOX is linked inside the pores of the MSNs [137] also protecting the drug. Hydrazone bonds have been added as hydrolysable bonds under acidic conditions opening the pores and releasing the drug. Biodistribution of such systems containing pH-sensitive opening cap pores (gates) in mice was also examined. These caps could be molecules with disulfide-linked bonds [138,139,140,141] or hydrolysable polysaccharides at low pH [142,143,144,145]. These systems have been designed toward the aim of retaining drugs in the pore until they are removed by external stimuli (pH, temperature, redox potential, light, and enzyme). Such a drug delivery carrier, based on rotaxane-modified MSNs, was investigated [146]. Multifunctional rotaxanes on MSNs were fabricated by using alkoxysilane tether, α-cyclodextrin (α-CD), and multifunctional peptides. The conjugated oligopeptides were composed of three functional segments, including a cell-penetrating peptide of seven arginine (R7) sequence, an enzyme-cleavable peptide of GFLG, and a tumour-targeting peptide of RGDS (Figure 5A). Incubating the DOX loaded MSNs with tumour and normal cells, the multifunctional nanoparticles could target tumour cells via the specific interaction between RGDS and integrin’s receptor αvβ3overexpressed on tumour cells, followed by penetrating the cell membrane with the aid of R7 sequence. After cellular uptake, drug-loaded MSNs released the encapsulated drug quickly due to the breakage of GFLG peptide cleaved by cathepsinB, resulting in enhanced antitumor activity (Figure 5A–F). This effective enzyme-responsive drug release system might have great potential in nanomedicine applications.

The stimuli-responsive behaviour can be achieved by grafting these moieties through cleavable bonds or using gatekeepers which suffer any chemical or physical change in response to different stimuli. These smart nanocarriers can transport the drug to the target tissue and once there, the presence of a certain stimulus, which may be internal like pH, redox, enzymes, small molecules, fusogenic lipids, etc., or external like light, temperature and magnetic field, will trigger the release of the trapped drugs achieving better control over the administered dose. Smart MSNPs that employ internal stimuli present the advantage of not requiring external apparatus to trigger the release. However, control over the administered dosage is lower than in the case of devices that employ an external stimulus. In another study it was reported that phenylboronic acid-functionalized MSNs can serve as an efficient co-delivery system for saccharide-responsive controlled release of insulin and cAMP [147].

### 2.4. Lipid-Based Nanoparticles (L-NPs)

The effective implementation of lipid-based nanoparticles (L-NPs) for carriage of molecules depends on their ability to penetrate some structural blockades, the sustained release of their drug content and their stability [148]. In the last decade, lipids have gained much interest as carriers for the delivery of drugs with poor water solubility [149]. L-NPs have the advantage of keeping the drug as a stable liquid solution, but the term ‘lipid formulation’ defines one of a large group of formulations [150]. Lipid nanoparticles have emerged as possible carrier systems to collect effectively the therapeutic profits of existing lipophilic molecules and new chemicals [151]. Lipids vary not only in structures and physiochemical properties, but also in their digestibility and absorption pathway; thus choice of lipid components and formulation type has a noticeable result on the biopharmaceutical characteristics of the drug molecule taken and circulation [152]. Moreover, either increasing or standardizing drug absorption is advantageous for poor therapeutic index molecules. Drug adsorption can be enhanced by using carriers which inhibit P-glycoprotein-mediated drug efflux and pre-absorptive metabolism involving gut membrane-bound cytochrome enzymes and promotion of lymphatic transport. These mechanisms deliver the active ingredient to the blood circulation system. In the same time, the hepatic first-pass metabolism is overcome and the gastrointestinal membrane permeability is improved [153,154]. During recent years, numerous materials have been loaded into these lipidic carriers, extending from lipophilic and hydrophilic molecules, with variable combinations, such as peptides and proteins [155,156,157].

#### 2.4.1. Solid Lipid Nanoparticles (SLNs)

**Figure 6:**
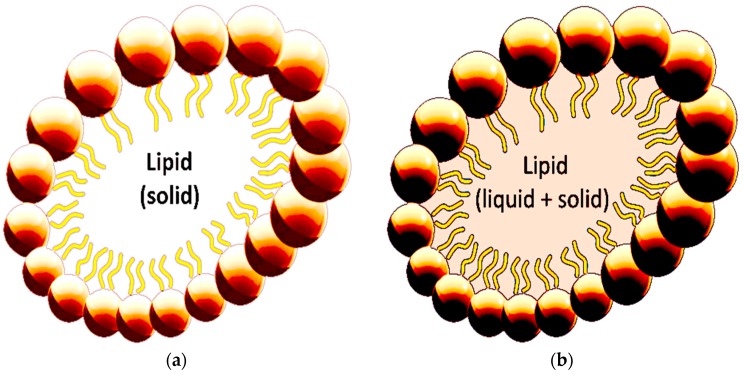
Structures of (**a**) solid lipid based nanocarriers (**b**) nanostructured lipid carriers.

The first SLNs were formulated in the early 90s as a different option for the researchers versus emulsions, liposomes, and polymeric nanoparticles in order to control the drug mechanism. SLNs contain a solid lipid (Figure 6a), where the drug is normally incorporated, with an average diameter below 1 µm [158,159]. The mentioned carriers involve a lipid matrix which is solid at room temperature and the average temperature of bodies. Alongside SLNs are biocompatible and biodegradable lipids that have also been applied as controlled drug delivery carriers for specific targeting. They are composed of a solid hydrophobic core having a monolayer coated by phospholipids while the solid part contains the drug dispersed or dissolved in lipid matrix [155,160]. SLNs are developed by abundant generally used methods such as hot homogenization, cold homogenization, microemulsion, solvent emulsification/evaporation, precipitation, *w*/*o*/*w* double emulsion, spray drying technique, etc. [160,161,162,163]. In addition, the common excipients used in SLN formulations are solid lipids, emulsifiers, co-emulsifiers and water [15,16]. They display significant advantages such as controlled release, increased bioavailability, defence of chemically changeable drugs, cost active excipients, improved drug loading and wide application [164]. The lipids used may be triglycerides, glycerides, fatty acids, steroids and waxes while a number of emulsifiers and their combinations have been used to optimize lipid dispersion. Combining emulsifiers may more effectively avoid particle accumulation [160,164]. Analogous with the surfactant used in the SLN, the oral absorption of the drug is differentiated because it can inhibit and deform the activity of the efflux transporters and the cell membrane [165]. The chemical structure of the lipid is imperative, because lipids which form highly crystalline particles with a perfect cage lead to drug dismissal throughout the packing period. Stable SLNs will be achieved only when the correct surfactant and regulated ratios have been operated [155].

Both hydrophobic and hydrophilic molecules such as paclitaxel, tobramycin, nifedipine, diazepam, desoxycorticosterone, hydrocortisone, doxorubicin, timolol, and pilocarpine have been loaded with SLN, and the application of SLN by different routes (parenteral, nasal, oral, ocular, topical etc.) has been examined [166].

#### 2.4.2. Nanostructured Lipid Carriers (NLCs)

The limitation of SLNs is that the full-crystallization or recrystallization of lipids can reduce drug solubility, preventing drug release from the SLNs, particularly when the amount of drug used during the process is too high. In a liquid lipid (oil), the drug solubility is greater in contrast to a solid lipid [167,168]. NLCs are the progressive generation of SLNs overcoming difficulties such as inadequate drug loading capacity, restructuring, and the expulsion of the drug in packing afterwards [169,170]. NLCs are carriers containing both solid lipid and oil as a core matrix (Figure 6b). Liquid lipids are better solubilizers of drugs than solid lipids [171]. NLCs possess various advantages for treatment over conventional systems, with improved solubility, the facility to improve storage stability, enhanced permeability and bioavailability, decreased adverse effects, prolonged half-life, and targeted tissue delivery [172]. Because of the physiological or biodegradable lipids, these carriers also shows a brilliant tolerability [173].

#### 2.4.3. Multifunctional SLNs and NLCs

Several factors play a huge role in designing a perfect nanocarrier. In fact, the surface charge interaction of the drug molecule and polymer or modification development are among the most important points. Surface modifications of lipid nanoparticles help to tune their properties to suit different applications in the field of nanotechnology. The modification improves the interfacial interactions between the particles and the lipidic matrix. Various researchers modified lipid nanoparticles with PEG, chitosan, biotin, peptide, Tween 80, d-α-tocopherylpoly(ethylene glycol 1000) succcinate, Eudragit RS 100, etc., [174,175].

Lipid nanoparticles can be functionalized with polymers or copolymers to make particles move towards the reticuloendothelial system. PEG is a coiled polymer of repeating ethylene ether units [176]. The stability of lipid can be improved by adding stabilizers such as propylene glycol, polyethylene glycol, especially for oral administration of these particulate systems [177]. There are a lot of US FDA-approved nanocarriers modified with PEG in preclinical studies for imaging, treatment, and drug delivery [178].

PEG stearate modified L-NPs have presented decreased uptake by macrophages in proportion to the length of the PEG chain after injection to animals intraperitoneally. Except this, SLNs are optimal nanocarriers for delivering drug to the brain because they have affinity and adhere to the endothelial cells of the blood–brain barrier [179]. The PEG modification of drug nanocarriers as it has been found extensively in the literature can hinder in vivo clearance by the mononuclear phagocyte system achieving an extended plasma half-life [180,181,182]. For cancer therapy, Wan et al. [183] developed vinorelbinebitartrate loaded PEG-modified SLNs. The vinorelbinebitartrate release study results showed faster drug release from PEG modified SLNs than unmodified SLNs. Cellular uptake results showed that the phagocytosis of PEG modified drug loaded SLN by RAW264.7 cells was inhibited effectively by the PEG modification of SLNs, while the uptake by cancer cells (MCF-7 and A549) could be enhanced significantly. Moreover, anticancer activity of vinorelbinebitartrate was considerably improved by the encapsulation of SLNs and PEG modified SLNs due to the improved cellular internalization of drug molecules.

NLCs can succeed passive targeting feature by changing particle size and obtaining active targeting properties by modification of character materials [184,185]. They can also be PEGylated to obtain long circulation [186]. Esposito et al. [187] prepared, characterized, and evaluated biodistribution of radiolabelled NLCs for in vivo tomographic examination. To this aim, a 99mTc complex was produced and incorporated in NLCs manufactured with an ultrasonication technique. NLCs were used for in vivo tomographic scanning of the rat body by a small-animal SPECT scanner that enabled the examination of NLC biodistribution after oral administration. It was seen when 99mTc-NLCs were directed orally, they were taken up by the gastrointestinal tract and transported to the reticulum-endothelial organs. This effect indicated that NLCs were rapidly cleared from the systemic circulation by opsonization and were uptaken by the reticulum-endothelial system.

Fuantes et al. [188] developed insulin loaded surface modified lipid nanoparticles to protect peptides within these carriers for oral drug delivery. The nanoparticles were modified with fatty acids such as poly(ethyethylene glycol)-2000 stearate and poly(ethylene glycol)-4500 stearate. PEG-stearate modified lipid nanoparticles were found to be more stable than unmodified carriers because their PEG modification protected aggregation in these mediums. In addition to this, they were significantly decreased pancreatin relevant degradation at 4 h.

In another recent study, Üstundağ Okur et al. [189] prepared nebivolol loaded SLNs with chitosan oligosaccharide lactate and polyethylene glycol stearate so as to improve the oral bioavailability of the drug. The release was identified as sustained without burst phenomenon while the enzymes’ presence seems to prevail the release. Beside this, SLNs were not cytotoxic and presented improved permeability via PEG modification.

Su et al. [190] investigated the impact of conjugated octreotide-polyethylene glycol monostearate for the improvement of targeting delivery of hydroxycamptothecine (HCPT) loaded in NLC. The in vivo pharmacokinetic study showed that the modified NLCs showed a longer circulation than NLC due to pegylation effect whereas the obtained results directed that the octreotide-polyethylene glycol monostearate highly modified NLCs would increase the effect of antitumor treatment by inhibiting the degradation, escaping RES and improving the hydroxycamptothecine uptake of tumour cells.

Luan et al. [186] developed baicalin loaded NLC which showed prolonged release and improved AUC compared to pure baicalin. Epidermal growth factor (EGF)-surface modified SLNs [191] were developed to deliver doxorubicin in the liver cancer model. EGF-SLN-DOX showed enhanced cytotoxic effect compared to SLN-DOX in both the cancer cell lines owing to the ligand targeted receptor mediated-endocytosis.

Peptide ligand modified SLNs were found to increase oral bioavailability of protein molecules. Peptide ligand modified SLNs loaded with salmon calcitonin (sCT), namely, sCT CSK-SLNs and sCT IRQ-SLNs, were developed by coupling the peptide ligand CSKSSDYQC (CSK) which was described to confirm attraction with goblet cells, or IRQRRRR (IRQ), a cell penetrating peptide, to polyoxyethylene (40) stearate (SA-PEG2000). CSK or IRQ modified SLNs with better CT safety capability could help the internalization of CT on Caco-2/HT29-MTX co-cultured cells and permeation. In conclusion, the lipid nanocarriers modified with CSK or IRQ peptide ligand could be possible formulations for the transference of peptide and protein molecules through intestinal walls [192].

Epidermal growth factor receptor (EGFR) targeting peptide-modified thiolated gelatin nanoparticles for *wild type-p53* gene delivery, delivery efficiency and transfection in pancreatic cells were evaluated so as to repair apoptosis and further trigger cell death in cancer cells. The system can work as a safe and efficient DNA delivery system for gene therapy and pancreatic cancer therapy [193].

Docetaxel loaded SLNs for oral anticancer therapy were conducted after the surface-modification of SLNs by Tween 80 or d-α-tocopherylpoly(ethylene glycol 1000) succinate (TPGS 1000). A sustained-release outline of drug from the SLNs was determined. Tween 80-emulsified SLNs presented improved intestinal absorption, lymphatic uptake, and oral bioavailability of drug compared with commercial formulations in in vivo studies [194].

Consequently, PEGylated carboxymethylcellulose modified NLCs (DTX-NLCs) for docetaxel delivery investigating the effect of the modification on in vitro and in vivo performances of NLCs. DTX-CNLCs showed better antitumor properties than unmodified DTX-NLCs in the tumour-bearing nude mice, owing to their long circulation property. Therefore, CNLCs hold great potential for actual delivery of anticancer molecules [195].

NLCs prepared using the melt-emulsification technique loaded with genistein were applied in recent ocular studies [196] showing interesting results. In fact, extended precorneal clearance and a 1.22-fold rise in AUC compared with the bare NLC as well as improved corneal penetration producing a 3.3-fold rise in permeability coefficients was observed.

Shah et al. [197] prepared modified NLCs for topical delivery so as to investigate the effect of polyarginine chain length on dermal delivery of surface modified NLCs. It was found that the surface modification of NLC with R11 boosted the transport of SP and KP across the skin barrier as well as decreasing inflammation reaction associated with ACD.

#### 2.4.4. Liposomes

Liposomes can be implied as promising carriers since several liposomic carriers are currently in clinical use due to their biodegradability and biocompatibility. Liposomes comprise of an aqueous core entrapped by one or more bilayers either as natural or synthetic lipids. They possess low toxicity natural phospholipids because they have weak immunogenicity. Liposomes can be applied so as to encapsulate both hydrophilic and hydrophobic molecules with the strongly lipophilic drugs being totally entrapped in the lipid bilayer while they are positioned entirely in the aqueous part. In addition, drugs with intermediate logP can perfectly be encapsulated in the middle of the lipid and aqueous phases [198,199].

As with the aforementioned NCs, liposomes have been functionalized over time in order to achieve desirable properties, like stability, cell penetration, and so on [200,201,202,203]. PEGylated liposomes as promising particles to achieve long-circulating or as effective drug carriers have been widely synthesized [204,205,206,207]. Liposomes modified with PEG encapsulating Tacrolimus also can act as a plausible candidate for cerebral ischemia-reperfusion injury [208]. PEGylated liposomes as well as folate-PEGylated liposomes containing paclitaxel were developed so as to decrease the cytotoxicity and improve the bioavailability and biocompatibility, acting as a targeting system cancer cell specific ligand folate. The results reveal that the folate-pegylated liposomes are ideal carriers for delivering Paclitaxel to the lymphatic system [209].

Liposomes containing PEGylated lipids which were linked covalently to oriented Annexin-A5 (Anx5) proteins were capable of targeting phosphatidylserine (PS)-exposing membranes given the high approachability of the reacting groups [210].

Among other challenges, crossing the blood–brain barrier is a complicated issue. Consequently, liposomes bi-functionalized with phosphatidic acid and a modified ApoE-derived peptide or an anti-transferrin receptor antibody, act as optimal candidates to cross the blood–brain barrier in vitro and in vivo [211,212]. Similarly, glutathione PEGylated liposomes were investigated as versatile carriers for enhanced drug delivery to the brain and ocular tissue [213,214]. Moreover, PEGylated doxorubicin in thermosensitive liposomes were formulated in order to differentiate the drug release decreasing tumour growth [215]. Lactoferrin considered as a target-ligand for hepatocellular carcinoma cells was applied as a modifying agent to PEGylated liposomes encapsulating doxorubicin, showing significantly strong antitumor efficacy [216].

**Figure 7:**
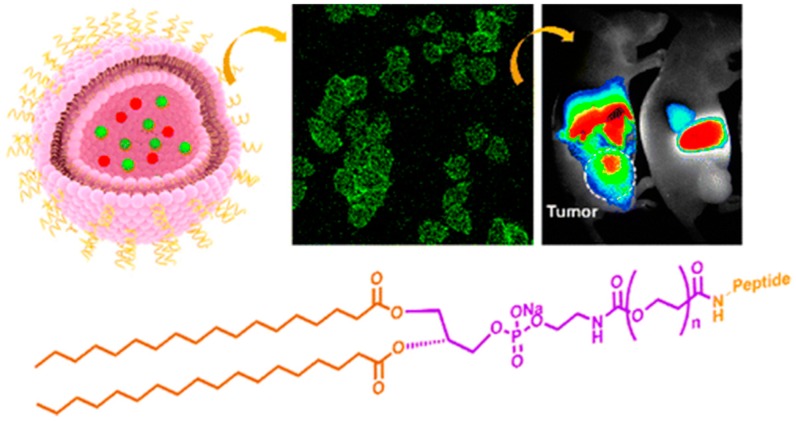
Schematic presentation of functionalized liposomes (pink sphere) with breast cancer targeting peptide (purple structure) and confocal images of liposomes (green spheres) in fluorescein isothiocyanate [217].

Functionalization of liposomes with a breast cancer targeting peptide (H6, YLFFVFER) as a targeting nanocarrier system (Figure 7) shows high efficiency since antitumor drugs were successfully delivered into human epidermal growth factor receptor 2 (HER2) positive breast cancer cells in both in vivo and ex vivo models [217]. Additionally, PEGylated liposomes modified with OX26 and chlorotoxin CTX were shown to promote cell transfection and improve the transport of plasmid DNA across the blood–brain barrier which further fights the brain glioma cells in vitro and in vivo [218]. The surface-modified pH-sensitive liposomal system was also shown to be a useful system for intracellular delivery of chemotherapeutics. Hyaluronic acid receptors conjointly with pH sensitive liposome carrier efficiently delivered Doxorubicin to the cancerous tissue by active targeting via HA and CD44 receptor interaction [219]. Likewise, the PEG-HA-modified liposomal siRNA delivery system with anti-γ-glutamylcyclotransferase (GGCT) siRNA proved to be an encouraging scenario in order to target MCF-7 breast cancer therapy due to retardation of tumour growth with negligible toxicity to healthy tissues [220]. Dual functionalized liposomes involving [D]-H_6_L_9_, a pH-responsive anti-microbial peptide (AMP), along with integrin αvβ3-targeted peptide (RGD) co-modified liposomes so as to improve tumour delivery efficiency. Paclitaxel-loaded liposomes further increase the cellular toxicity against C26 cancer cells compared with liposomes modified only with RGD and [D]-H6L9, respectively, revealing an intriguing tumour inhibition effect [221].

### 2.5. Dendrimers

Another essential category of multifunctional NCs which have gained the attention of researchers are dendrimers. Owning their name to the Greek word “dendro-tree”, dendrimers are nano-sized structures consisting of tree-like arms or branches. They were first discovered between the late 70s and early 80s. Dendritic macromolecules play an emerging role in anticancer strategies and diagnostic imaging materials. Due to their peripheral groups which are capable of modifications with antibody, peptides or proteins, dendrimers are adept at hosting several molecules [12,222].

**Figure 8:**
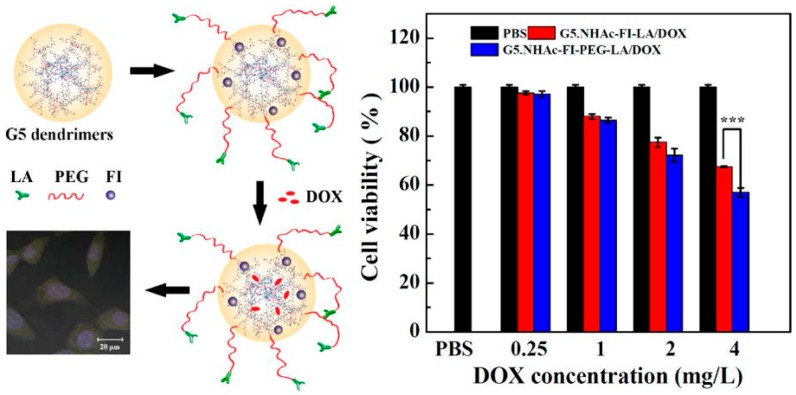
Multifunctional lactobionic acid-modified dendrimers for targeted drug delivery to liver cancer cells [223].

Liver targeted systems are actually a challenging issue for pharmaceutical technologists. It was reported that a dendrimer system that was modified by lactobionic acid (LA) targeted cancer cells which overexpress asialoglycoprotein receptors. Additionally, the used doxorubicin drug was dismissed in a sustained manner (Figure 8) [223]. Phosphorylated PAMAM dendrimers were found to be more suitable to be employed in the human body in contrast with unmodified ones while they have great potential in dentistry clinical treatments [224].

Triazinedendrimers derivatized with paclitaxel were found to be optimal for increasing the aqueous solubility of the anticancer agent. In addition, this carrier was further pegylated showing possibilities for anticancer strategies [225]. Another similar triazinedendrimer conjugated with paclitaxel and PEG moieties seems to persist in the vasculature longer whereas it showed higher tumour uptake [226].

POXylated polyurea-dendrimers are efficiently synthesized expressing fluorescence, pH-responsiveness and improved aqueous solubility in the form of core-shell smart nanocarriers. These materials are satisfactory transfection agents, using an endosomal pathway, which reduces the IC50 of paclitaxel [227].

PAMAM dendrimer-modified laponite nanodisks were used to encapsulate doxorubicin with an exceptional tremendous loading efficiency displaying a pH-dependent sustained release profile, delivering doxorubicin in an acidic environment. They were able to effectively be uptaken by cancer cells showing a stronger inhibitory effect in contrast to free DOX [228].

Trifunctionaltriazine derivatives containing folic acid and methotrexate were coupled in order to produce bifunctional dendrimer therapeutics which induced higher cytotoxicity in KB tumour cells [229]. Moreover, a monovalent folic acid was conjugated with a PAMAM dendrimer leading to improved binding between the folic acid–polymer conjugate and a folate binding protein surface [230]. Furthermore, PAMAM dendrimer conjugated with folic acid was used as substrate-triggered dendrimer binding to a bovine folate binding protein exosite. Researchers suggest that this technique is promising for binding scaffolds containing drugs along with imaging agents to desired protein targets [231].

Among these strategies, the use of modified chitosan linked with PAMAM dendrimers showed a different property, as an antimicrobial agent. More specifically, a novel water soluble quaternizedcarboxymethylchitosan (core) and poly(amidoamine) dendrimer (PAMAM) shell were reported as core-shell nanoparticles showing strong antibacterial activity [232]. Another group notified that combining CS and PAMAM dendrimer indicated better hemocompatibility whereby safer and more effective drug and gene delivery vehicles are possible [233]. Zwitterionic chitosan with a unique pH-sensitive profile was also utilized so as to modify the surface of PAMAM dendrimers in order to eliminate haemolytic and cytotoxic phenomena from the red blood cells and fibroblast cells from PAMAM dendrimers, respectively, while penetration of PAMAM dendrimers in cells was also allowed. Its pH responsiveness demonstrates that coated PAMAM dendrimers can be used as a candidate to target solid tumours which present microenvironments [234].

Hyaluronic acid grafted [235] and conjugated [236] PAMAM dendrimers for prolonging the systemic circulation and actively targeting the tumour and delivery of topotecan hydrochloride and 3,4-difluorobenzylidene curcumin pancreatic cancer therapy were evaluated, respectively, with promising results.

### 2.6. Polymeric NPs-NCs

Over the past three decades, polymers have been explored as key components in systems delivering active ingredients, vitamins, peptides or as imaging sensors. They are organic chemical substances composed of repeated units called “monomers” whereby they are mainly categorized as synthetic or natural. Macromolecules should present biocompatibility and histocompatibility, hydrolytic degradation producing non-toxic monomers as well as other chemical properties in order to be characterized as optimal candidates in pharmaceutical technology [237,238]. NCs consisting of polymers can be applied in several forms, such as nanoparticles, nanocapsules, nanofibers and nanogels. Several drugs have been loaded in such systems presenting high loading capacity, sustained or immediate release rate and biological properties.

#### 2.6.1. NCs Derived from Natural Polymers

Polymers such as chitosan, alginic acid, hyaluronic acid, etc. occur in nature and are widely used as biomaterials, drug delivery systems and even in imaging diagnostics. Their interesting structures with active groups (hydroxyl, amino, carbonyl) can easily be modified with other polymers, sugar moieties, peptides, proteins, etc. in order to induce pH or temperature responsiveness, targetability, and mucoadhesiveness [239,240,241].

#### 2.6.2. Chitosan NCs

**Figure 9:**
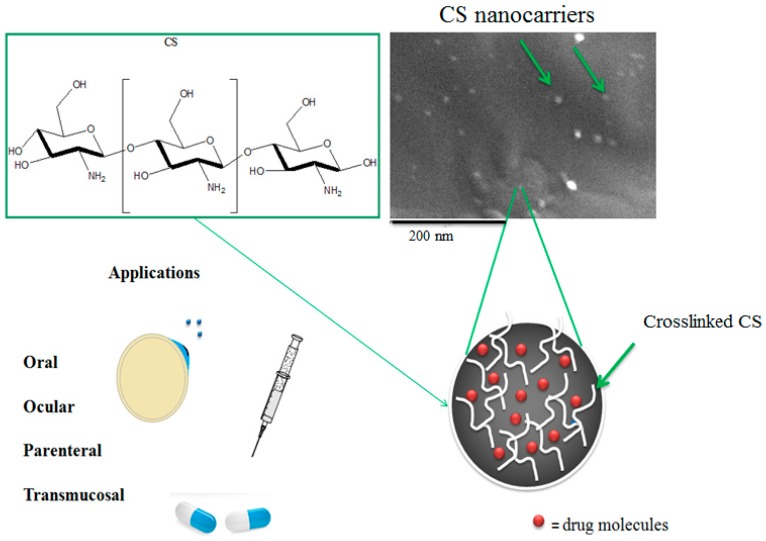
CS nanocarriers for several medical and pharmaceutical applications.

Chitosan (CS) is a natural cationic polysaccharide derived from the exterior skeleton of crustacean shells. CS presents haemostasis, biocompatibility, mucoadhesiveness, fungistatic and antimicrobial properties, which makes this macromolecule a primary material in drug delivery systems [18,242,243,244]. Moreover, CS, due to the active groups (hydroxyl and amino groups) found on its structure, can be easily modified, producing multifunctional materials for cancer therapy, and ocular, oral and parenteral systems. Multifunctional NCs from this polysaccharide have been used in several applications (Figure 9). Its cationic structure provides the opportunity to self-structured when CS linked with negative groups, into NPs. Multifunctional CS NPs via ionic gelation have been used as targeted systems due to their expressed mucoadhesiveness. According to Siafaka et al. [242], nanocarriers of *N*-succinyl and *N*-Carboxybenzyl chitosan derivatives were prepared via ionotropic gelation for the loading of Timolol while their size ranged between 190 and 525 nm. Due to their higher swelling ability, the release was found to be sustained in most cases. Jing and co-workers have developed multifunctional nanoparticles which were covalently modified with ureido-conjugated chitosan (UCCs) as a targeted delivery system for *Helicobacter pylori* elimination. *Helicobacter pylori* growth was restrained on account of the encapsulation of amoxicillin into the specific UCCs/TPP nanoparticles compared with unmodified amoxicillin-chitosan nanoparticles [245]. HA coated CS NPs using ionic gelation had lower cytotoxicity compared to CS-NPs [246].

Folic acid is a receptor expressed to cancerous cells. Consequently, folate targeted systems from CS-folate-PEG have shown to improve cell targeting and tumour penetration [247]. In addition, magnetic CS nanoparticles loaded with bleomycin drug were reported as a new, smart nanotherapeutic system for cancer therapy [248]. Chitosan-based nanoparticles comprised of pure chitosan and *O*-carboxymethyl chitosan were debilitated in multilayer sodium alginate beads for oral delivery of anticancer drug doxorubicin presenting ideal properties [249]. Glycol chitosan nanoparticles with hydrotropic 2-(4-(vinylbenzyloxy)-*N*,*N*-diethylnicotinamide) oligomers have been reported as a new tumour targeting system of paclitaxel delivery system, improving the solubility of PTX [250]. Additional, synthesized nanoparticles of peptide-tagged (PEG)ylated chitosan were used as promising carriers in neurodegenerative disorders for siRNA delivery [251].

#### 2.6.3. Hyaluronic Acid (HA)

HA, also called hyaluronan, is an anionic, non-sulfated polymer belonging to polysaccharides composed of d-glucuronic acid and d-*N*-acetylglucosamine, linked via β-1,4 and β-1,3 glycosidic bonds. Due to its cyto and histo-compatibility, HA is broadly used in the form of nanocarriers either as a coating layer or as self-assembled nanoparticles. Improved intravitreal mobility for nonviral polymeric gene nanomedicines, specifically p(CBA-ABOL)/pDNA complexes, after HA coating has been reported [252]. Moreover, amphiphilic HA-5b-cholanic acid conjugates (HA-CA conjugates) were prepared via chemical conjugation of hydrophobic bile acid (5b-cholanic acid) and hydrophilic HA backbone expressing active tumour targeting [253]. However, a main drawback of hyaluronic acid (HA)-based drug conjugates or nanoparticles, as anticancer strategies, is their great accumulation in the liver. PEGylated-HA NPs were synthesized in order to prolong the systemic circulation supporting high tumour targetability [254]. Cholesterylhemisuccinate modified HA nanocarriers loaded with Docetaxel were developed in order to target passive and active tumour tissues [255,256]. A multifunctional delivery system based on cholesterol-conjugated HA micelles was developed in order to encapsulate Doxorubicin and superparamagnetic nanoparticles for cancer therapy [257]. Multifunctional NPs consisting of HA-α-tocopheryl succinate conjugate and d-α-tocopheryl polyethylene glycol succinate loaded with docetaxel were also studied as targeted systems in cancer therapy revealing positive results [258]. Among these applications, tumour-targeted NPs systems for MR imaging of tumours are in high demand. Modifying Fe_3_O_4_ NPs with HA, the formed multifunctional Fe_3_O_4_ NPs showed water dispersion, stability, and cytocompatibility and haemocompatibility as well as efficiency as nanoprobes for specific MR imaging [259,260]. HA functionalized uniform mesoporous carbon nanoparticles, increasing colloidal stability, biocompatibility, cell-targeting ability, and controlled release [261].

#### 2.6.4. Alginic Acid and Its Salts

Alginic acid, also known as algin or alginate, is an anionic polymer found widely in the cell walls of brown algae presenting homopolymeric blocks of (1,4)-linked β-d-mannuronate (M) and its C-5 epimer-α-l-guluronate (G) residues, covalently linked together. It is widely used due to its mucoadhesive properties. Borges et al. demonstrated that the sodium alginate-coated nanoparticles’ system presented lower burst release of loaded ovalbumin whereas it was an effective adjuvant for hepatitis surface antigen when administered in mouse [262]. DNA-loaded chitosan nanoparticles modified with alginic acid against breast cancer demonstrated a noticeable trend as pH values increased [263]. Alginate–gelatin nanoparticles were evaluated as the simplest desirable delivery vehicle due to their prolonged drug release profile and the ECM proliferation which they induce [264].

#### 2.6.5. NCs of Synthetic Polymers

NPs from synthetic polymers such as aliphatic polyesters are widely used in commercial pharmaceutical products due to their important properties such as biocompatibility, biodegradability as well as active groups (−OH) which can covalently link or conjugate with several receptors so as to improve cellular uptake and increase the circulation time to RES, while their encapsulation efficacy was found to be extremely high. Poly(lactic acid), poly(ε-caprolactone) and poly(lactic acid-co-glycolic acid) were examined several decades ago while polyesters of succinic acid are attracting the interesting of researchers nowadays [265]. Among their advantages, the expressed hydrophobicity of these polymers leads to massive clearage by the macrophages of the phagocytic system. Thus, the surface of polymeric nanoparticles is modified with various molecules improving the circulation time and persistence in the blood [266].

Recently, novel polymeric nanoparticles consisting of biodegradable and biocompatible polymers have been used, encapsulating active biomolecules. Nonetheless, the difficulty of administering the drug clinically limits its use greatly. Recently, copolymers made from aliphatic polyesters such as PLA and PCL and attached with hydrophilic segments (via “PEGylation” or “POXylation”) reveal interesting results in terms of increasing the blood circulation time as well targeting the desirable tissues. A notable aspect of such polymers is their interaction with biological systems given that they are expected to be well tolerated (i.e., to be biocompatible) with low toxicity, protein interaction, unspecific organ accumulation and low to no immunogenicity. Poly(2-oxazoline)s have shown great haemocompatibility, low cytotoxicity and improved stability in comparison to poly(ethylene glycol) (PEG) segments.

Poly(ε-caprolactone) is an aliphatic polyester which is biocompatible and biodegradable, classifying it as a favourable carrier in pharmaceutical technology. However, PCL presents high crystallinity affecting drug loading, release pattern and targetability [267]. Poly(lactic acid) is also an aliphatic polyester derived from lactic acid monomers while its copolymerization with glycolic acid produce poly(lactic acid-co-glycolic acid) copolymers. FDA (Food and Drug Administration) classified these polymers as a Generally Regarded as Safe (GRAS) substance [268]. Their bio- and cytocompatibility seems to play a major role in choosing such polymers as nanoparticulate drug delivery systems. Moreover, they present the facility to be modified by easy steps whereas coating also may differentiate their characteristics. PEGylation, POXylation or HAylation are procedures which are utilized so as to enhance the cells’ permeability by the addition of hydrophilic segments on their backbones. HAylation, the covalent conjugation of HA to bioactive molecules, can help to overcome drawbacks related with some pharmaceuticals, such as insolubility, instability and fast kidney clearance. Nevertheless, nanoparticle targeting of tumour cells is improved using targeting moieties, e.g., antibodies or antibody fragments, peptides and sugars, due to the receptor’s expression in cancer cells [269].

Poly(ethylene glycol) is a hydrophilic polymer highly recognized as a biomaterial which provides hydrophilicity via coupling or conjugation methods [270]. Poly(2-oxazolines) belonging to the pseudopolypeptides are known as biocompatible substances which, although having important characteristics, have not been assigned the significance which they deserve [271].

Multifunctional poly(ethylene glycol)-*block*-poly(lactic acid) (PEG-*b*-PLA) or poly(lactic acid-co-glycolic acid) (PEG-*b*-PLGA) [272,273,274] and poly(ethylene glycol)-block-poly(ε-caprolactone) (PEG-*b*-PCL) [275,276,277,278] nanoparticles have been designed for cell targeting, imaging, retention of EPR effect, penetration, better biocompatibility as well as controlled release for several years now. Novel strategies involve the use of peptides or proteins and other moieties for promoting their possibilities.

It has been reported that PLA NPs are unstable in gastrointestinal (GI) fluids, due to the protonation of carboxylic acid groups in low pH, promoting its precipitation whereas PEG-b-PLA NPs were found to be stable in such low pH [279]. Furthermore, methoxy poly(ethylene glycol)-poly(l-histidine)-poly(lactide) (mPEG_45_-PH_30_-PLA_82_) triblock copolymers self-assemble into nanoparticles by stereocomplexation indicating that the stereocomplexation between PLLA and PDLA was able to prevent the aggregation of the nanoparticles at pH values close to 6.8 [280]. Recently, a polyelectrolyte ionomer complex (PIC) comprised of cationic and anionic polymers particularly, a poly(ethylene glycol)-poly(lactic acid)-poly(ethylene imine) triblock copolymer (PEG-PLA-PEI) and a poly(aspartic acid) (P[Asp]), enabled colloidal stability while doxorubicin (DOX) loaded PIC demonstrated pH-dependent behaviour and anticancer properties [281].

Other studies have shown that pegylated PLA NPs moved more easily to the intestine developing significant interactions with the mucosa, reaching the epithelium. The aforementioned mucus permeation was more acute for nanoparticles coated with PEG2000 or PEG6000 than with PEG10000 [282]. The same group recently evaluated the use of Pluronic covered PLA NPs so as to overcome the low chemical and colloidal stability in the gastrointestinal track for PLA nanoparticles [283].

Biodegradable, pH-sensitive poly(methacrylic acid)-block-poly(lactic acid)-block-poly(methacrylic acid) (PMAA_2_-b-PLA-b-PMAA_2_) type multiblock copolymers have successfully been synthesized as a promising carrier of paclitaxel (PTX) for intestine cancer-targeting therapy with sustained release and high safety [284]. Stimuli-responsive nanocarriers from poly(d,l-lactide)-*block*-poly(2-aminoethyl methacrylate) (PLA-*b*-PAEMA) were further modified by 2,3-dimethykmaleic anhydride (DMMA) and succinic anhydride (SA)loaded with DOX demonstrated long systemic circulation, improved tumour–cell adhesion, tumour targeted drug release as well as tumour cells cytotoxicity after the charge-conversion [285].

Respectively, a dual-targeted pH-sensitive biocompatible polymeric nanosystem was prepared using ATRP-based biodegradable triblock copolymer, poly(poly(ethylene glycol) methacrylate)-poly(caprolactone)-poly(poly(ethylene glycol) methacrylate) (pPEGMA-PCL-pPEGMA) and it was examined as a potential mechanism against various epithelial cancers. Dual targeting achieved with folate and the AS1411 aptamer improved the cancer-targeting efficiency of the nanoparticles which were loaded with DOX, resulting in enhanced cellular uptake [286].

Multifunctional pH-sensitive nanoparticles consist of poly(lactic acid)-poly(ethylene glycol)-poly(l-lysine)-diethylenetriaminepentaacetic acid (PLA-PEG-PLL-DTPA) and the pH-sensitive poly(l-histidine)-poly(ethylene glycol)-biotin (PLH-PEG-biotin) was developed for concurrent tumour magnetic resonance imaging (MRI) and anticancer therapy using the anti-hepatocellular carcinoma (HCC) drug sorafenib. They reported that the target pH-sensitive theranostic nanoparticles were produced after the chelation of the Gd ions to the DTPA groups, and the conjugation of biotinylated vascular endothelial growth factor receptor (VEGFR) antibodies was conjugated to the surface biotin groups of nanoparticles [287].

**Figure 10:**
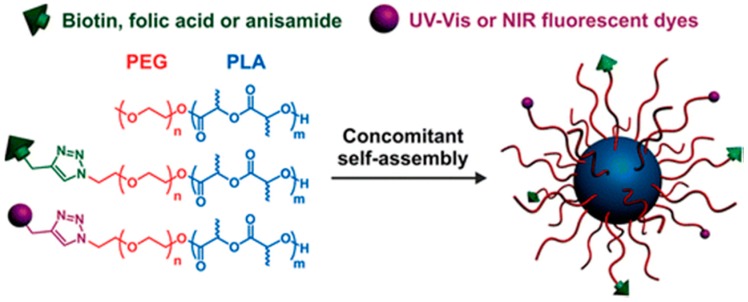
Multifunctional poly(ethylene glycol)-block-poly(lactic acid) (PEG-*b*-PLA) nanoparticles for cancer cell targeting and imaging [288].

Nanoparticles containing a fluorescent probe along with a targeting ligand were synthesized involving PLA-*b*-PEG-ligand, PLA-*b*-PEG–fluorescent probe, as well as PLA-*b*-PEG-OMe copolymers. Similarly, authors suggested that various multifunctional nanoparticles can be prepared by decoration with biotin, folic acid or anisamide, fluorescent nanoparticles (UV-vis or near-infrared dyes) (Figure 10) [288].

Moreover, pH-responsive docetaxel-conjugated poly (lactic acid) PLA-(PEG)-folate micelles were prepared to carry docetaxel (DTX). This pH-responsive nanoconjugate system revealed different release properties analogous with the acidic or neutral environment [289]. PEG-PLA micelles bound with cell penetrating decapeptide arginine-glycine (RG)_5_ and a pH-sensitive decapeptide histidine-glutamic acid (HE)_5_ formulations revealed that the drug was low internalized presenting release at basic pH [290].

A star-shaped folate-core polylactide-d-α-tocopheryl polyethylene glycol 1000 succinate (FA-PLA-TPGS-DM1) copolymer was loaded with emtansine (DM1) in order to achieve great anticancer activity both in vitro and in vivo in breast cancer models in comparison with linear FA-PLA-TPGS nanoparticles [291]. In a similar manner, PLGA nanoparticles functionalized with cholic acid and TPGS were applied so as to deliver docetaxel to cervical cancer [292]. PLA–TPGS nanoparticles conjugated with folic acid which were further loaded with paclitaxel display great advantages compared to free PTX. The folate conjugation significantly enhances the targeted delivery of drug to both in vitro and in vivo cancer cell models [293].

Nanoparticles comprised of PEGylated PLA and PLGA loaded with oleanolic acid were found to express enhanced cytotoxicity against cancer cells whereas PLGA formulation has a greater ability to kill the cancer cells [294]. In addition, reverse nano-micelles of *b*-cyclodextrin (CD) conjugated with PEG-*b*-PLA were employed as BSA protein delivery system with great success [295].

Further conjugation of PEG-PLA/PLGA/PCL with Hyaluronic acid had different effects in the systems. Hyaluronic acid-decorated PLGA-PEG nanoparticles were utilized in order to effectively target ovarian cancer [296]. Similarly, in the past w HA-PEG-PCL NPs were developed for doxorubicin delivery [297]. PLGA, Pluronic F127 (PF127), chitosan, and (HA) were composed as dual NPs with characteristics suggesting that they were optimal candidates to co-deliver multiple anticancer drugs against the drug resistance mechanisms of cancer stem-like cells [298].

Different blocks of (poly(ε-caprolactone)_2_-[poly(2-(diethylamino)ethyl methacrylate)-*b*-poly(poly(ethylene glycol) methyl ether methacrylate)]_2_ [(PCL)_2_(PDEA-b-PPEGMA)_2_] micelles were used to encapsulate DOX considering their pH-responsiveness. It was found that the in vitro DOX release from the micelles was greatly improved by eliminating pH from 7.4 to 5.0. [299]. Furthermore, poly(ethylene glycol)-*b*-polycaprolactone (PEG-PCL) copolymer micelles with size control synthesized via a membrane contactor method were used to efficiently encapsulate the hydrophobic drug (Vitamin E) providing pH-dependent properties [300].

Novel stealth nanoparticles of poly(ethylene glycol)-block-poly(ε-caprolactone)-and phospholipid-based were applied as PTX nanocarriers showing enhanced therapeutic effectiveness on murine breast cancer due to the improved intracellular drug delivery [301]. Additionally, PTX was encapsulated in novel lipid–polymer hybrid drug nanocarriers consisting of folate (FA) modified lipid-shell-1,2-distearoyl-sn-glycero-3-phosphoethanolamine-*N*-[methoxy (polyethylene glycol)-2000] DSPE-PEG2000 (as lipid-shell), and PCL-PEG-PCL (as self-assembled core) (FLPNPs) expressing controlled and targeted delivery of PTX inhibiting tumour growth [302]. It was refereed that methoxy-poly(ethylene glycol)-*b*-polycaprolactone (mPEG-b-PCL) and folate-functionalized PEG-b-PCL were developed so as to be engulfed by ARPE-19 (human RPE cell line) via receptor-mediated endocytosis. Findings supported that the binding affinity and the uptake was of the greatest extent when the folate loading into the NPs was 100% [303]. Similarly, mPEG-b-PCL polymer micelles were decorated with FA in order to actively target cancer lines whereas the additional super paramagnetic iron oxide (SPIO) resulted in tremendous delivery efficiency and excellent imaging improvement [304]. PTX loaded PCL-TPGS nanoparticles exhibited longer blood circulation time and a slower plasma elimination rate than the commercial products Taxol(^®^) and Abraxane(^®^) proving that TPGS decoration was significant [305].

Novel micelles like nanocarriers of Poly(2-ethyl-2-oxazoline)-PLA-g-PEI amphiphilictriblock were applied for the co-delivery of minicircle DNA and chemotherapeutics exhibiting suitable characteristics for both in vitro and in vivo administration of minicircle DNA with high efficacy and negligible cytotoxicity [306]. Oral chemotherapy of lung cancer was achieved via thiolated chitosan-modified PLA-PCL-TPGS-encapsulated PTX nanoparticles due to its mucoadhesiveness and permeation properties [307].

**Figure 11:**
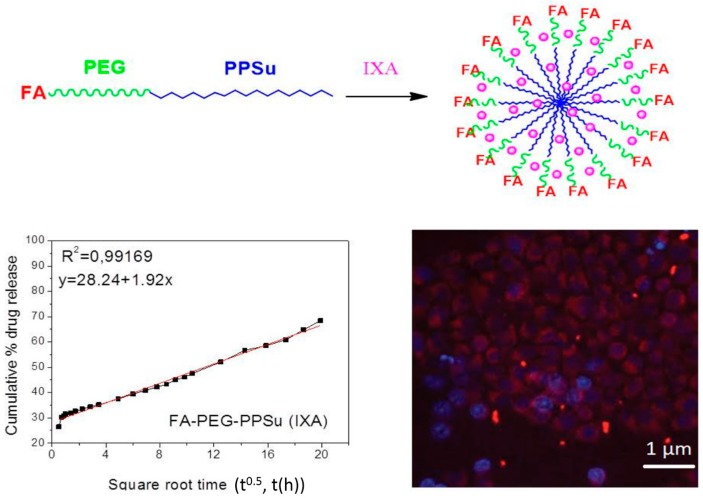
Synthesis of folate-pegylated polyester nanoparticles encapsulating ixabepilone (red spheres) for targeting folate receptor overexpressing breast cancer cells (blue cells) [2].

It can be mentioned that many studies for PLA, PCL and PLGA polymers can be found every year for several applications, mainly in cancer therapy. Notwithstanding, a variety of polymers have gained the attention of researchers for biomedical nanoapplications. In fact, poly(propylene succinate) (PPSu) is a new biocompatible aliphatic polyester with a low melting point (T_m_ = 44 °C), close to the physiological body temperature. Pegylated PPSu nanoparticles based on PEG–PPSu copolymers have been developed recently as therapeutics loaded with cisplatin, ropinirole and tibolone [308,309]. A novel folate system of pegylated PPSu (Figure 11) was used to deliver a new anticancer drug known as Ixabepilone showing promising properties [2]. Another group used the nanoprecipitation method in preparing multifunctional NPs to produce nanoparticles of a 30–200 nm size consisting of the biodegradable polyesters poly(butylene sebacate) (PBSeb) and poly(butylene sebacate-*co*-butylene dilinoleate)s. This size average enabled the NPs’ internalization by various cell lines [310].

Polyurethanes (PUs) are synthetic polymers linked with urethane (or carbamate) bonds (–NH–COO–) in their main chains. They have been functionalized in order to tailor favourable features while they have been put forward as drug delivery systems [311]. Temperature- and pH-responsive nanoparticles of biocompatible polyurethanes were found suitable for doxorubicin delivery [312] whereas polyesterurethane (PU) based nanoparticles (NPs) containing indomethacin were embedded in a scaffold so as to be applied as a local delivery system [313]. Moreover, some authors suggested that amino-functionalization of biodegradable polyester- and polyester/ether-urethanes (PURs) NPs can actively recognize target cells [314].

## 3. Future Aspects

Over the past three decades, nanocarriers have attracted the attention of research groups from all over the world in order to manufacture formulations against several diseases. Although there has been a huge improvement in pharmaceutical nanotechnology so as to limit undesirable problems induced from NPs, still some issues should be addressed. First of all, the most novel nanotherapeutics are only currently subject to experiments. For instance, the cytotoxicity studies of the multifunctional NCs are limited to the cell series of specific types while some of them are applied in animal models. Consequently, the most promising nanosystems have to undergo clinical studies. In association with this, the clinical trials must be well designed in order to cover a broad area of diseases. Additionally, the cytotoxicity experiments and clinical studies have to be conducted over several years so as to determine possible limitations or side effects. For example, coated inorganic materials which are new in the nanomedicine field must be studied for both short- and long-term safety.

## 4. Conclusions

Nanoparticles, due to their lower size, permeability as well as enhanced drug loading ability as a result of the higher surface volume ratio, are ideal platforms for drug delivery. Their features, mainly their toxicity and targetability, can be improved via surface functionalization. In such a way, carbon nanomaterials, silica nanoparticles, lipid, polymeric and dendritic nanocarriers are widely coated or conjugated with folic receptors, proteins and hydrophilic or hydrophobic segments preparing multifunctional and stimuli responsive nanoplatforms. Analogous with the used carrier, different properties can be aroused such as release pattern, specific targeting, biocompatibility, and other advantageous properties. From a clinical perspective, nanocarriers exist already in the market but, given the diverse strands of research emerging with new nanosized agents being published every day, it can be concluded that nanotechnology represents the future.
